# SOCS6 promotes radiosensitivity and decreases cancer cell stemness in esophageal squamous cell carcinoma by regulating c-Kit ubiquitylation

**DOI:** 10.1186/s12935-021-01859-2

**Published:** 2021-03-12

**Authors:** Xuanzi Sun, Yuchen Sun, Jing Li, Xu Zhao, Xiaobo Shi, Tuotuo Gong, Shupei Pan, Zhongqiang Zheng, Xiaozhi Zhang

**Affiliations:** 1 Department of Radiation Oncology, The First Affiliated Hospital of Xi’an Jiaotong University, No. 277, Yanta West Road, Xi’an, 710061 Shaanxi China; 2 Department of Radiation Oncology, The Second Affiliated Hospital of Xi’an Jiaotong University, Xi’an, Shaanxi China; 3 Department of General Surgery, The First Affiliated Hospital of Xi’an Jiaotong University, Xi’an, Shaanxi China

**Keywords:** Esophageal squamous cell carcinoma, Radiosensitivity, SOCS6, Cancer cell stemness, c-Kit, Ubiquitylation

## Abstract

**Background:**

Radiotherapy is a major treatment for esophageal squamous cell carcinoma (ESCC). However, HPV infection related radioresistance caused poor prognosis of ESCC. The function of SOCS6, which has been shown to be a tumor suppressor in several cancers, has not been fully investigated up till now. In this manuscript, we aim to further investigate the role of SOCS6 in regulating ESCC radioresistance.

**Methods:**

Fifty-seven ESCC patients were enrolled for survival analysis. SOCS6 was stably overexpressed in HPV^+^ ESCC and ESCC cells, and cells were treated with radiation and then subjected to colony formation assays. Expression of DNA damage repair regulating proteins were examined by Western blotting. Cell growth, cell migration and cisplatin sensitivity were then analyzed. Sphere formation assays and flow cytometry were used to investigate changes in cancer stem cell (CSC) properties. Immunofluorescent staining and confocal microscopy were used to locate SOCS6 and c-Kit. Ubiquitylation level of c-Kit were analyzed after immunoprecipitation. Then, coimmunoprecipitation (CoIP) of SOCS6 and c-Kit were performed. In vivo, xenograft animal models were treated with radiation to examine the radiosensitivity.

**Results:**

SOCS6 is correlated with better prognosis in ESCC patients. Radioresistance is impaired by SOCS6 upregulation, which inhibited cell growth, migration and increased sensitivity to cisplatin. SOCS6 significantly decreased the population of CSCs expressing the surface biomarker CD271 or CD24^low^/CD44^high^ and their ability of sphere formation. SOCS6 and c-Kit were collocated in the cytoplasm. Blotting of ubiquitin and CoIP experiments indicated that the mechanism was related to ubiquitylation and degradation of the receptor c-Kit. Xenograft tumor mouse model showed that SOCS6 inhibited tumor growth and promoted radiosensitivity in vivo.

**Conclusions:**

Our findings suggest that SOCS6 can promote the radiosensitivity of HPV^+^ ESCC and ESCC cells and reduce their stemness via ubiquitylation and degradation of c-Kit. Thus, SOCS6 is a potential target for overcoming radioresistance of ESCC.

**Supplementary Information:**

The online version contains supplementary material available at 10.1186/s12935-021-01859-2.

## Background

Esophageal cancer is a highly malignant type of cancer and is the seventh leading cause of cancer death worldwide [1]. The two major subtypes of esophageal cancer are esophageal adenocarcinoma (EAC) and esophageal squamous cell carcinoma (ESCC). ESCC accounts for approximately 90% of all cases of esophageal cancer worldwide, and 79% of ESCC cases happen in Central and Southeast Asia [2, 3]. Among cases in Asia, more than half occurs in China. Radiotherapy (RT) is the major treatment for ESCC. Despite advances in surgical techniques and optimization of chemoradiotherapy protocols, the prognosis of ESCC is poor, with 5-year overall survival (OS) rates ranging from 20 to 35% [4]. A dominant cause of the low OS rate is acquired radioresistance. Radiosensitivity is known to be affected by microbial infection, such as human papillomavirus (HPV). HPV is a major prognostic factor in head and neck squamous cell carcinoma (HNSCC) and cervical cancer [5]. HPV positive HNSCC patients tend to have better prognosis [6]. In ESCC, our previous study found that HPV is a negative prognostic factor and that HPV attenuates the radiosensitivity of ESCC cells [7] .

The suppressor of cytokine signaling (SOCS) family consists of eight proteins, namely SOCS1-7 and cytokine-inducible SH2-containing protein (CIS), which are known for their negative regulation of cytokine receptor signaling [8]. Among the eight members, SOCS1 and SOCS3 are well characterized. SOCS1 regulates cell proliferation in breast cancer, hepatocellular carcinoma, thyroid cancer and ESCC, while SOCS3 exerts effects in gallbladder cancer, gastric cancer and ovarian cancer by inhibiting JAK2/STAT3 signaling [9–15]. SOCS6 is known to suppress tumor growth in multiple cancers such as gastric cancer, prostate cancer, non-small cell lung cancer and cervical cancer, through regulating tumor angiogenesis and cell apoptosis [16–19]. SOCS6 also participates in renal fibrosis, diabetic retinopathy, multiple sclerosis and other non-neoplastic diseases [20–22]. Moreover, as part of the Cullin-Ring ligase 5 complex, SOCS6 mediates the ubiquitination and degradation of receptor tyrosine kinases (RTKs) via its SOCS box domain [23]. Insulin receptor substrate 4 (IRS4), FMS-like tyrosine kinase 3 (Flt3) and c-Kit have been predicted to be targets of SOCS6 [24–26]. SOCS6 has also been demonstrated to recruit and degrade YAP, JAK2 and Sin1 proteins through the ubiquitin-proteasome system (UPS) [27–29].

Cancer stem cells (CSCs) have been indicated to possess self-renewal and differentiation ability, which leads to radioresistance, tumor metastasis and recurrence [30]. The surface markers CD271 (also named p75NTR) and a CD24^low^/CD44^high^ phenotype have been reported to be biomarkers for human esophageal CSCs [31]. C-Kit (also named CD117) is a proto-oncogene that is either mutated or upregulated in numerous cancers, such as lung cancer, acute myeloid leukemia (AML) and gastrointestinal stromal tumor (GIST) [32, 33]. Preclinical research has shown that a humanized anti-c-Kit antibody is a promising treatment for c-Kit-positive cancers [34]. In addition, c-Kit is a CSC marker, and inhibition of c-Kit reduces the stemness of cancer cells [35, 36] .

We previously reported that downregulation of SOCS6 via ceRNA mechanism increased cell growth of ESCC and that HPV infection increased the proportion of CSCs in ESCC cells, which induced radioresistance [37, 38]. Our current study illustrated that SOCS6 played a tumor-suppressing role in ESCC. We reported herein the function of SOCS6 in regulating radioresistance and CSCs through ubiquitination of c-Kit in ESCC.

## Methods

### Cell culture

Eca109 and KYSE-150 cells were cultured in RPMI-1640 medium supplemented with 10 % FBS, and 2mM  l-glutamine at 37 °C, in a 5% CO_2_ atmosphere in a humidified cell incubator. For overexpression of SOCS6, cells were transfected with lentiviruses. Lentiviruses overexpressing full-length SOCS6 were purchased from Genechem (Shanghai, China). For knockdown of SOCS6, short hairpin RNAs (shRNA) designed to target SOCS6 (shRNA sequence: GCAGAAGGGAAGCTAGCAACTCGAGTTGCTAGCTTCCCTTCTGC, GCACTCAAATGGTAGGTTTCTCGAGAAACCTACCATTTGAGTGC) were obtained from Genechem.

### Cell proliferation assay

Cell proliferation was evaluated with a WST-8 [2-(2-methoxy-4-nitrophenyl)-3-(4-nitrophenyl)-5-(2,4-disulfophenyl)-2H-tetrazolium Sodium Salt; Abcam, US] assay. Two thousand cells in 100 µL of medium were seeded in 96-well plates, and were then cultured for the indicated numbers of days. Then, 10 µL of WST-8 solution was added to the plates and incubated for 4 h. The optical density (OD) of each well was measured every 30 min using a microplate reader at 460 nm. For the drug-sensitivity assay, 4000 cells were seeded, and a WST-8 assay was performed following the same method.

### Colony formation assay

Eca109 and KYSE-150 cells were seeded in six-well plates and irradiated with gradient doses (0, 2, 4, 6 and 8 Gy; Clinac 2100EX X-ray linear accelerator, Varian Medical Systems) of X-rays. After 10–14 days of cell growth, during which the cell media were changed every 2–3 days, cells in the six-well plates were fixed with 4% paraformaldehyde, stained for 6 min at room temperature with 0.5% crystal violet dissolved in 25% methanol, and washed with running water. Colony formation was documented by photography, and colonies were counted in ImageJ. The survival fraction (SF) was calculated, according to which survival curves were fitted using Single-hit multi-target model SF = 1 − (1−e^− kD^)^N^ by GraphPad Prism 8. The radiobiological parameters (D_0_, D_q_, N, SF_2_) were generated based on survival curves.

### Western blot analysis

Cells were lysed with radioimmunoprecipitation assay (RIPA) lysis and extraction buffer (Pioneer Technology, Xi’an, China). Proteins were separated by SDS-PAGE on 10–15% Tris-glycine gels and electrophoretically transferred to PVDF membranes (Millipore, Billerica, MA, USA). Membranes were then blocked in 5% nonfat milk and incubated overnight at 4 ℃ with the following monoclonal antibodies: anti-SOCS6 (ab197335, Abcam, 1:500), anti-c-Kit (#3074; Cell Signaling Technology (CST), Beverly, MA, USA; 1:1000), anti-Ku70 (#4588; CST, 1:1000), anti-Ku80 (#2753; CST, 1:1000), anti-RAD51 (#8875; CST, 1:1000), anti-p-ATR (#2853; CST, 1:1000), anti-HPV16 E6 (ab70; Abcam, 1:500), anti-CD24 (18330-1-AP, Proteintech, 1:500), anti-CD44 (15675-1-AP, Proteintech, 1:500), and anti-GAPDH (10494-1-AP, Proteintech, 1:1000). Membranes were washed four times in TBST and then incubated with HRP-conjugated anti-rabbit (1:20,000) or anti-mouse (1:20,000) secondary antibodies (CST) for 1 h at room temperature. Membranes were washed again, and immunoreaction were visualized using a chemiluminescence reagent (Millipore, Billerica, MA, USA) and a ChemiDoc System (Bio-Rad, Hercules, CA, USA). GAPDH was used as the internal reference protein to ensure equal loading.

### Transwell migration assay

Transwell chambers in 24-well plates were purchased from Corning (Corning, NY, USA). Two hundred microliters of serum-free medium containing 1 × 10^5^ cells was added to the upper chambers. Then, 600 µL of medium supplemented with 20% FBS was added to the lower chambers as an attractant. Cells were cultured for 24 h and washed with PBS. Cells remaining on the upper surface of the chamber membranes were wiped off with a cotton swab. Cells that migrated to the bottom surface of the chamber membranes were fixed with 4% formaldehyde and stained with 0.5% crystal violet. Cells were counted in five random photographed fields.

### Sphere formation assay

Cell suspensions were seeded in ultra-low-attachment 96-well plates (Corning) at concentration of 200 cells per well in serum-free-medium (DMEM/F12) supplemented with B27, basic-FGF and EGF. Cells were cultured for 10–14 days, during which medium was added every 2–3 days. On the last day of the experiment, 10 µL of trypan blue dye solution was added to each well to exclude dead cells, and the formed spheres were counted.

### Immunofluorescence

Immunofluorescence was performed as previously described [38]. Cells were incubated with antibodies: anti-SOCS6 (#3074; Santa Cruz Biotechnology, Santa Cruz, CA, USA, 1:200), anti-c-KIT (#3074; CST, 1:200).

### Immunoprecipitation (IP)

Eca109 and KYSE-150 cells cultured in 10-cm dishes were lysed with 3 mL of ice-cold RIPA buffer, and the lysate was centrifuged at 10,000×*g* for 10 min to pellet debris. One microgram of normal rabbit IgG was added to the lysate with 20 µL of Protein A/G PLUS-Agarose (Santa Cruz). The beads were pelleted by centrifugation at 2500 rpm for 5 min, and the supernatant was collected. A rabbit monoclonal anti-c-Kit antibody (CST, 1:200) was added and incubated for 1 h at 4 ℃. Twenty microliters of Protein A/G PLUS-Agarose were added and incubated at 4 ℃ on a rocker overnight. After 4 washes with RIPA buffer and centrifugation, the pellet was resuspended in 40 µL of 1× electrophoresis sample buffer and boiled prior to SDS-PAGE.

### Ubiquitylation assay

SOCS6 overexpressing Eca109 and KYSE-150 cells and control cells were treated with MG132 (20 µg/mL) for 4 h before IP with the rabbit monoclonal anti-c-Kit antibody (CST, 1:200). Ubiquitylation was assessed using a mouse monoclonal anti-Ub antibody (Santa Cruz, P4D1, 1:100).

### Flow cytometry analysis

About 1 × 10^6^ cells were collected and washed with ice-cold PBS. Then, 100 µL of PBS and 20 µL of an anti-CD271-PE antibody alone or a combination of anti-CD24-PE and anti-CD44-APC antibodies were added to the cells and incubated for 30 min. Then, the samples were washed twice and analyzed using a FASCCalibur MT flow cytometer (BD Bioscience, USA). For all samples, the number of events were set to 10,000 counts.

### Xenograft tumor model and treatment

Female BALB/c nude mice (4 weeks old) were purchased from the Experimental Animal Center of the School of Medicine (Xi’an Jiaotong University). Eca109 cells (1 × 10^6^ cells/100 µL) with stable SOCS6 expression or control cells were injected subcutaneously into the inguinal region of each mouse. Mice were weighed and tumor sizes were measured every 4 days. The tumor volume was calculated according to the following formula: volume = (a × b^2^)/2, where a is the longest diameter of the tumor and b is the perpendicular diameter. After 28 days, mice were sacrificed, and tumors were isolated and embedded in paraffin for immunohistochemical (IHC) staining analysis. To verify the radiosensitivity of ESCC cells, Eca109 cells (1 × 10^6^ cells/100 µL) with stable SOCS6 expression or control cells were injected subcutaneously into the inguinal region of each mouse. When the tumor volume reached 100 mm^3^, the mice were treated with 2 Gy of radiation targeted to the tumor region and were kept alive for 2 more weeks before sacrifice and tumor excision. The tumor size was recorded. The animal experiments were approved by the Institutional Animal Ethics Committee of the First Affiliated Hospital of Xi’an Jiaotong University, and all experiments were conducted in accordance with the Animal Ethics guidelines of the First Affiliated Hospital of Xi’an Jiaotong University.

### IHC staining analysis

IHC staining analysis was conducted as described previously [22]. The primary antibodies used were anti-SOCS6 (ab197335, Abcam, 1:100) and anti-c-Kit (#3074, CST, 1:100) antibodies.

### Statistical analysis

All data are presented as the mean ± standard deviation (SD) of values from at least three independent experiments. Statistical analysis was performed using GraphPad Prism 8 (GraphPad Software Inc., San Diego, CA). The significance of differences between the groups was evaluated by the Student’s t-test. A value of *P* < 0.05 was considered statistically significant.

## Results

### SOCS6 promotes radiosensitivity in HPV^+^ ESCC and ESCC cells

**Fig. 1:**
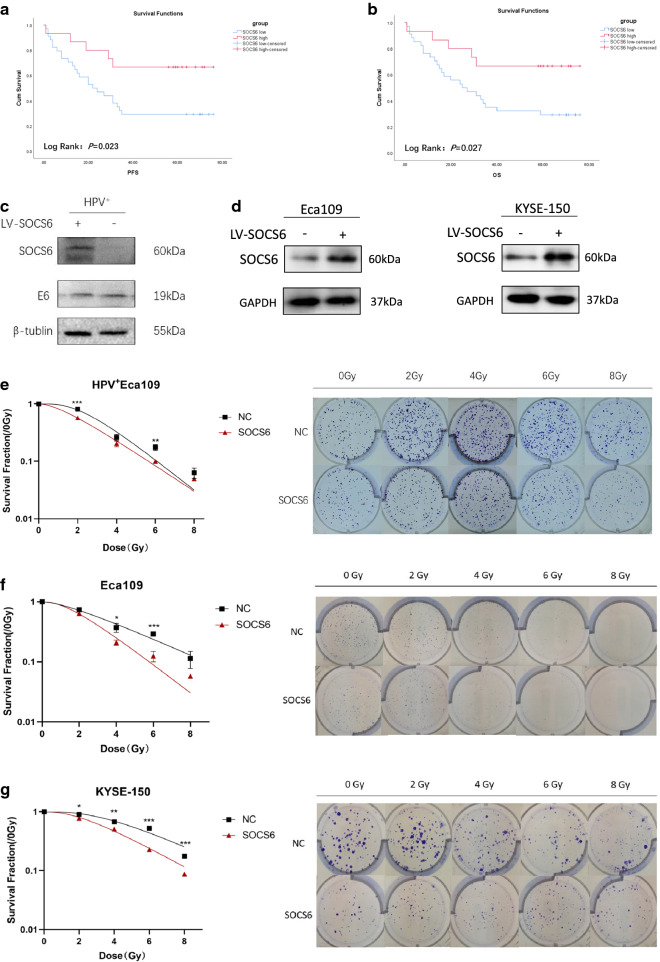
SOCS6 expression is correlated with prognosis of ESCC patients and radiosensitivity of ESCC cells. **a**, **b** Effects of SOCS6 expression on survival of 57 ESCC patients (red: SOCS6 high; blue: SOCS6 low); **c**, **d** ESCC cells were transfected with lentivirus expressing the SOCS6 protein, and SOCS6 expression was validated by WB analysis (n = 3); **e**–**g** cells overexpressing SOCS6 were treated with 0, 2, 4, 6, or 8 Gy of 4 MV X-rays (n = 3). Cells were stained and counted after 14 days of culture. The survival curves are shown on the left. The data are presented as mean ± SD, *P < 0.05, **P < 0.01 and ***P < 0.001

Expression data in Oncomine database showed that SOCS6 expression in ESCC tissue is significantly lower than that in normal esophageal tissue (Additional file 1: Fig. S1a, *P* = 6.56 × 10^− 5^; 1b, *P* = 1.66 × 10^− 15^). Fifty-seven ESCC patients were included for survival analysis. Patients with high SOCS6 expression presented better progression-free survival (PFS, *P* = 0.023, Fig. 1a) and overall survival (OS, *P* = 0.027, Fig. 1b). Since low expression of SOCS6 is correlated with poor prognosis in ESCC patients, we suspected that SOCS6 is a negative regulator of ESCC and that SOCS6 might promote radiosensitivity. Previous study found that HPV infection is also correlated with poor prognosis in ESCC patients. Therefore, HPV^+^ Eca109, KYSE-150 and Eca109 cells were transfected with lentivirus expressing the SOCS6 protein, and SOCS6 expression was evaluated by Western blotting (WB) analysis (Fig. 1c, d). HPV E6 onco-protein was also detected by WB (Fig. 1c). We first examined whether overexpression of SOCS6 could increase the radiosensitivity of HPV^+^ ESCC cells. The results of colony formation assays showed that SOCS6 promoted radiosensitivity of HPV^+^ Eca109 cells (Fig. 1e, *P* < 0.01). Survival curves were generated by one target and one-hit model. There was a significant left-downward trend of survival curve in SOCS6 overexpressing cells. Colony formation assays were also performed on Eca109 and KYSE-150 cells and survival curves were fitted. Results showed that ESCC cells overexpressing SOCS6 were more sensitive to radiation than the corresponding control cells (Fig. 1f, g, *P* < 0.05). The radiobiological parameters (D_0_, D_q_, N, SF_2_) of SOCS6 overexpressing group were lower than those of the control cells (Table 1, *P* < 0.05). Therefore, it can be concluded that SOCS6 promotes radiosensitivity in HPV^+^ ESCC and ESCC cells.

**Table 1:** Radiobiological indices of the SOCS6-overexpressing cells and control cells

	D_0_	D_q_	N	SF_2_
HPV^+^				
NC	1.612 ± 0.038	1.980 ± 0.061	4.496 ± 0.193	0.812 ± 0.026
SOCS6	1.925 ± 0.071**	1.305 ± 0.058***	1.920 ± 0.130***	0.574 ± 0.019***
Eca109				
NC	3.145 ± 0.118	1.663 ± 0.172	1.704 ± 0.374	0.738 ± 0.062
SOCS6	1.838 ± 0.059***	1.478 ± 0.138	2.409 ± 0.401	0.638 ± 0.050
KYSE-150				
NC	2.732 ± 0.496	2.680 ± 0.391	4.098 ± 0.476	0.905 ± 0.020
SOCS6	2.593 ± 0.133	1.919 ± 0.052*	2.639 ± 0.256**	0.776 ± 0.014*

*P < 0.05, **P < 0.01 and ***P < 0.001

**Fig. 2:**
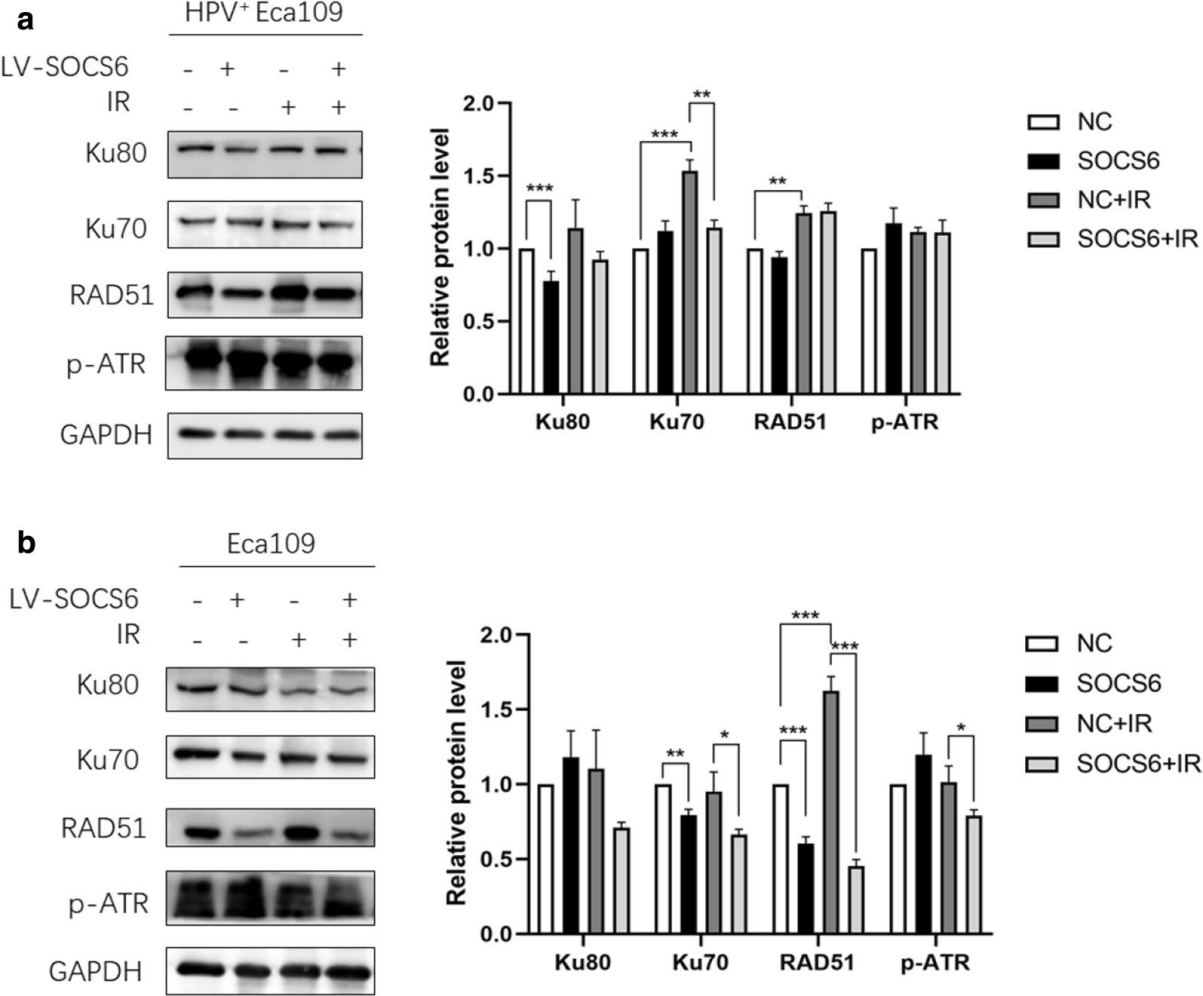
NHEJ and HR are involved in the promotion of ESCC radiosensitivity. **a** HPV^+^ Eca109 cells with or without SOCS6 overexpression were given 4 Gy of X-ray before subjected to WB of HR and NHEJ proteins; **b** Eca109 cells with or without SOCS6 overexpression were given 4 Gy of X-ray before subjected to WB of HR and NHEJ proteins (n = 3). The data are presented as mean ± SD, *P < 0.05, **P < 0.01 and ***P < 0.001

Radiosensitivity is regulated by several mechanisms such as DNA damage repair, cell cycle arrest, cancer cell stemness and microbial infection. Nonhomologous end-joining (NHEJ) and homologous recombination (HR) are two essential DNA damage repair pathways. HPV^+^ Eca109 and Eca109 cells were given 4 Gy of X-ray before WB of key regulators in NHEJ and HR were performed. In HPV^+^ Eca109 cells, SOCS6 overexpression decreased Ku80 (*P* < 0.001) expression, while Ku70 (*P* < 0.01) decreased after irradiation (Fig. 2a). In Eca109 cells, SOCS6 overexpression decreased the expression of Ku70 (*P* < 0.01) and RAD51 (*P* < 0.001), while Ku70 (*P* < 0.05), RAD51 (*P* < 0.001) and p-ATR (*P* < 0.05) decreased after irradiation (Fig. 2b). These results suggest that suppression of Ku70, Ku80, RAD51 and ATR by SOCS6, which lead to inhibition of both NHEJ and HR, are involved in the mechanism of ESCC radiosensitivity.

### SOCS6 inhibits cell growth and migration and improves sensitivity of Cisplatin of ESCC cells

**Fig. 3:**
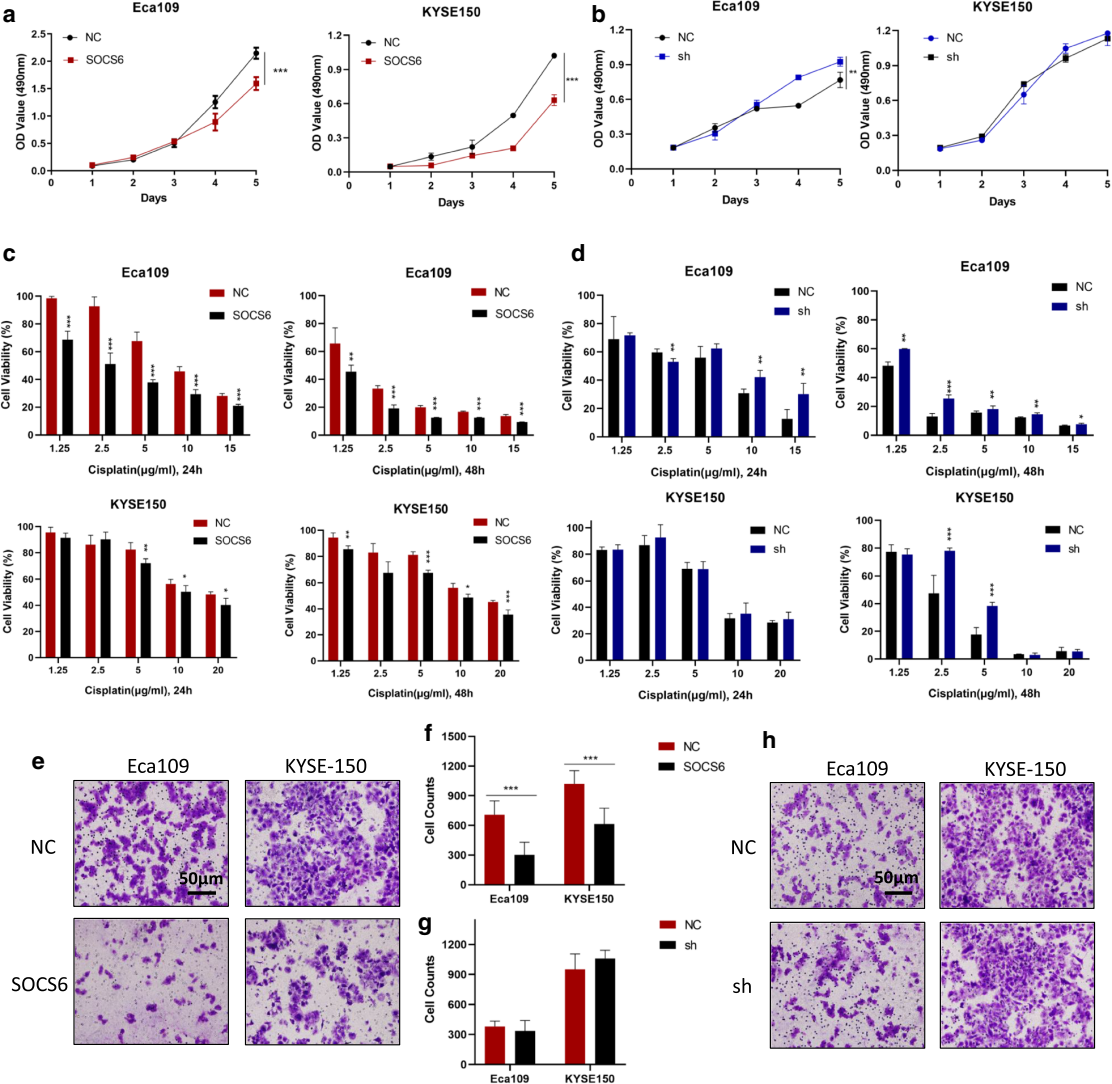
SOCS6 suppresses cell proliferation, migration and drug resistance of ESCC cells. **a**, **b** Cell proliferation assays were performed, and SOCS6 was found to inhibit cell growth (n = 5); **c** Eca109 and KYSE-150 cells were treated with cisplatin (1.25, 2.5, 5, 10 and 15 µg/mL for Eca109 cells and 1.25, 2.5, 5, 10 and 20 µg/mL for KYSE-150 cells) for 24 h and 48 h respectively (n = 5). Cisplatin sensitivity increased significantly with overexpression of SOCS6; (d) Cisplatin sensitivity decreased with knockdown of SOCS6 (n = 5). **e**, **f** The migration ability of Eca109 and KYSE-150 cells with SOCS6 overexpression was evaluated by transwell assays, which indicated that SOCS6 decreased cell migration ability (n = 3). **g**, **h** Knockdown of SOCS6 did not impact cell migration ability (n = 3). The data are presented as mean ± SD, *P < 0.05, **P < 0.01 and ***P < 0.001

To determine whether SOCS6 can inhibit malignant behaviors of ESCC cells, cell growth, migration and drug sensitivity assays were performed. The results of the WST-8 assays showed that the proliferation rate was significantly decreased in Eca109 and KYSE-150 cells overexpressing SOCS6 compared to the corresponding control cells (Fig. 3a, *P* < 0.01). Then, Eca109 and KYSE-150 cells were treated with graded concentrations of cisplatin for five days, and cell viability was evaluated. The viability of cells overexpressing SOCS6 was decreased, indicating that SOCS6 can significantly increase cisplatin sensitivity in ESCC cells (Fig. 3c, *P* < 0.05). The effect of SOCS6-knockdown was also studied. Knockdown of SOCS6 promoted cell growth in Eca109 cells (Fig. 3b, *P* < 0.01). However, it did not significantly alter cell growth in KYSE-150 cells (Fig. 3b, *P* = 0.42). As for cisplatin sensitivity, knockdown of SOCS6 decreased Eca109 and KYSE-150 cells’ sensitivity to cisplatin at some concentrations (Fig. 3d, *P* < 0.05). To investigate the effect of SOCS6 on ESCC cell motility, cell migration was evaluated after overexpression and knockdown of SOCS6 in ESCC cells. In transwell assays, the migration ability of ESCC cells stably overexpressing SOCS6 was decreased (Fig. 3e, f, *P* < 0.001), whereas cells with knockdown of SOCS6 did not alter cell motility (Fig. 3 g, h, Eca109: *P* = 0.43, KYSE-150: *P* = 0.20). These results suggest that SOCS6 suppresses malignant behaviors of ESCC cells such as proliferation, migration and drug resistance.

### SOCS6 suppresses cancer cell stemness of HPV^+^ ESCC and ESCC cells

**Fig. 4:**
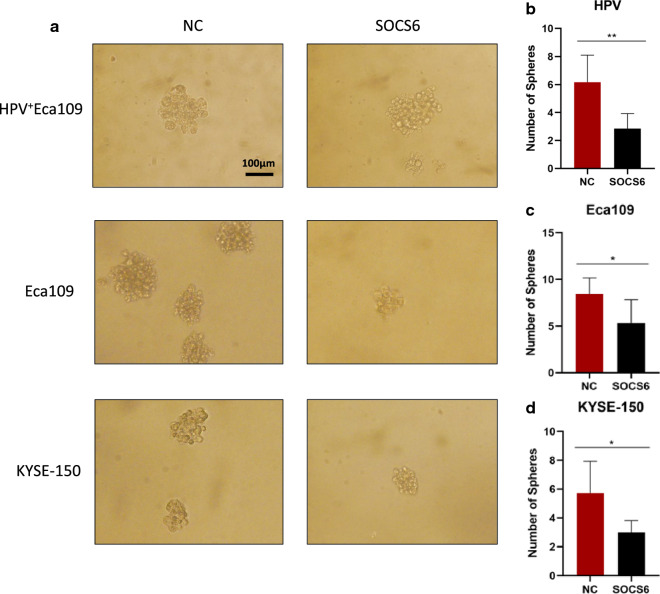
SOCS6 decreases the sphere formation ability of ESCC cells. **a** Cells were cultured in serum-free medium for 10–14 days to allow the formation of CSC-like spheres. The sphere formation ability was significantly decreased in cells overexpressing SOCS6. Representative photographs are shown; **b**–**d** quantification of spheres (n = 6). The data are presented as mean ± SD, *P < 0.05, **P < 0.01 and ***P < 0.001

Since cancer cell stemness is considered an essential factor of radioresistance, we further investigated whether SOCS6 expression affects the stemness of ESCC cells. Sphere formation assays were performed to investigate the changes in cell stemness due to alterations of SOCS6 expression. In HPV^+^ Eca109 cells, cell spheres formed in SOCS6 overexpression group was significantly reduced compared with the control group, which means cancer cell stemness was attenuated (Fig. 4a, b, *P* < 0.01). The number of spheres formed by Eca109-SOCS6 and KYSE-150-SOCS6 cells was obviously decreased compared with the number formed by the corresponding control cells on day 14 (Fig. 4a, c, d, *P* < 0.05).

**Fig. 5:**
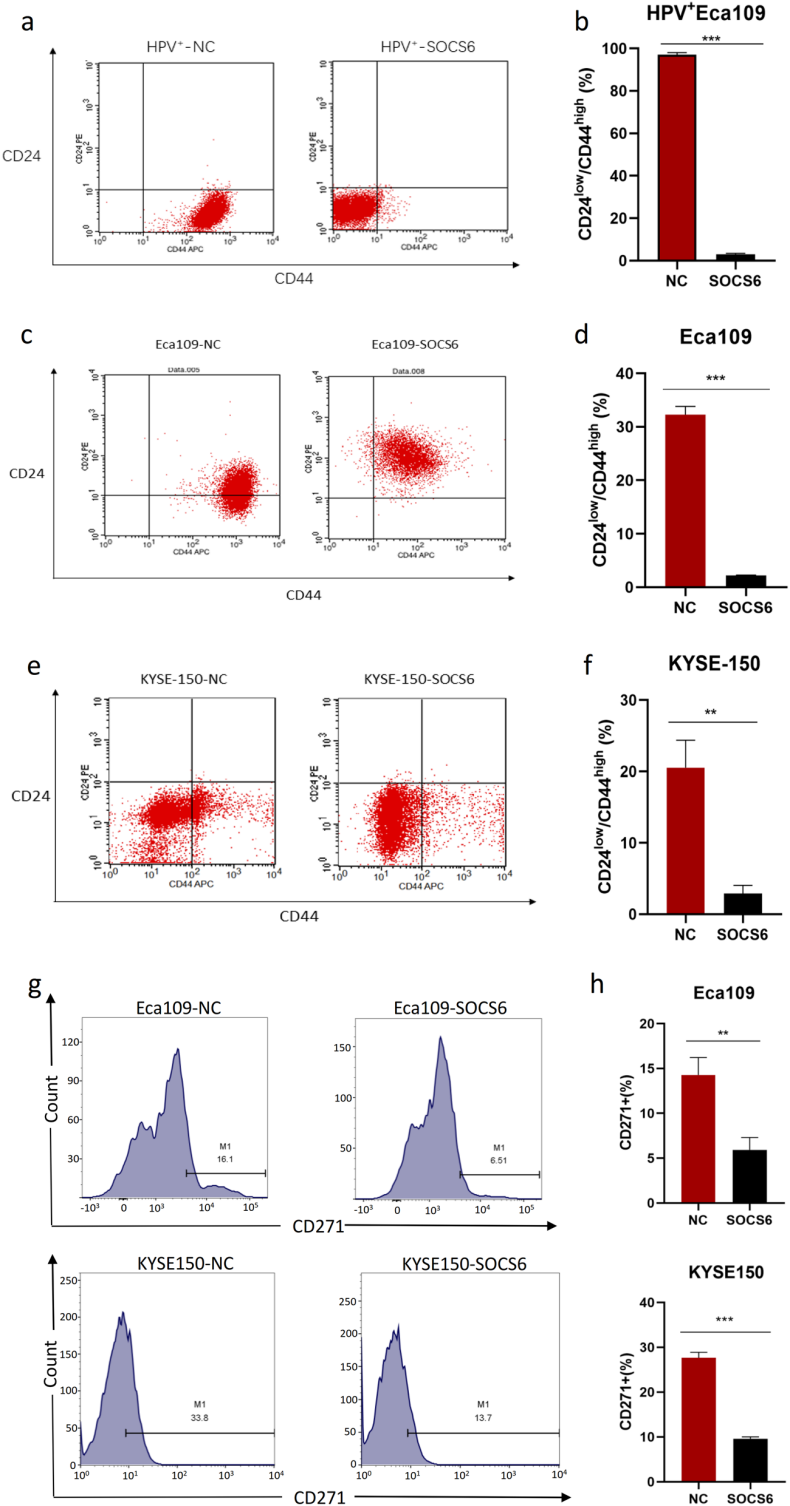
SOCS6 is a negative regulator of ESCC cell stemness. **a** HPV^+^ Eca109 cells were analyzed by flow cytometry after incubation with antibodies specific for CD24/CD44 for 30 min; **b** the statistical results are shown (n = 3); **c**, **e** Eca109 and KYSE-150 cells were also analyzed by flow cytometry for CD24/CD44 (n = 3). SOCS6 overexpression significantly decreased the population of CD24^low^/CD44^high^ cells; **d**, **f** the statistical results are shown (n = 3); **g** Eca109 and KYSE-150 cells were analyzed by flow cytometry for CD271. SOCS6 significantly decreased the population of CD271^+^ Eca109 and KYSE-150 cells. **h** The statistical results are shown (n = 3). The data are presented as mean ± SD, *P < 0.05, **P < 0.01 and ***P < 0.001

We used flow cytometry to examine the expression of cancer stem cell-related surface markers. CD24^low^/CD44^high^ is the surface marker phenotype of CSCs, and CD271 is an ESCC stem cell-specific marker. After exogenous expression of SOCS6 in HPV^+^ Eca109 cells, CD24 expression increased significantly while CD44 expression decreased, indicating loss of cell stemness (Fig. 5a, b, *P *< 0.001). Similarly, the proportion of CD24^low^/CD44^high^ cells decreased in SOCS6 overexpression group in Eca109 (*P* < 0.001) and KYSE-150 (*P* < 0.01) cells (Fig. 5c–f). The population of CD271^+^ cells was also decreased in both Eca109 (*P* < 0.01) and KYSE-150 (*P* < 0.001) cells overexpressing SOCS6 (Fig. 5g, h). Taken together, these results indicate that SOCS6 is a negative regulator of ESCC cancer cell stemness, a role that may account for the increases in radiosensitivity of ESCC cells overexpressing SOCS6.

The population of CD271^+^ cells increased significantly in Eca109 cells after radiation of 8 Gy of X-rays (Additional file 2: Fig. S2a, b, *P* < 0.05). This result is consistent with other studies [5]. However, SOCS6 overexpression suppressed the induction of the CSCs phenotype (Additional file 2: Fig. S2a, b, *P* < 0.001). This result provides further support to explain the increased radiosensitivity in SOCS6-overexpressing cells.

### SOCS6 binds with c-Kit and promotes its ubiquitylation

**Fig. 6:**
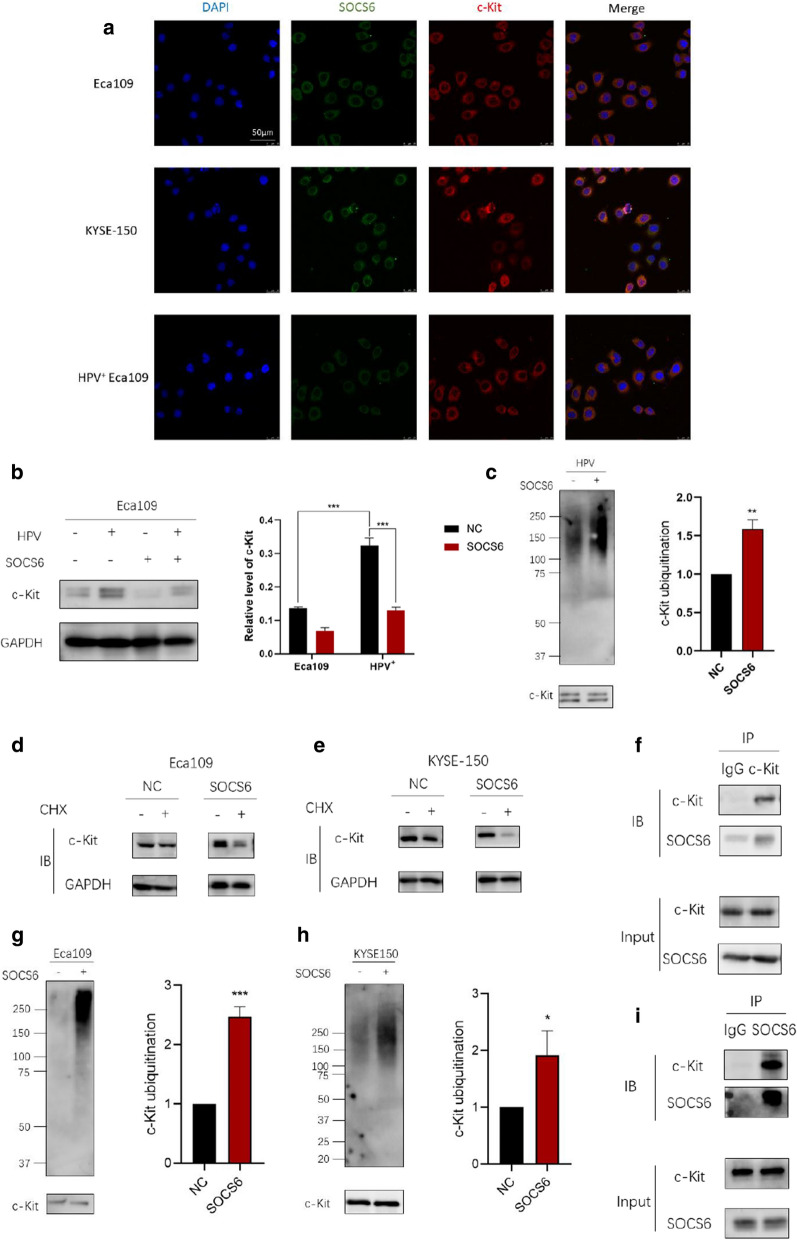
SOCS6 regulates the ubiquitylation of c-Kit. **a** SOCS6 and c-Kit were immunofluorescent stained and analyzed under laser scanning confocal microscopy; **b** the expression of c-Kit was detected by WB. In cells overexpressing SOCS6, c-Kit expression was down regulated; **c** in HPV^+^ Eca109 cells, c-Kit was immunoprecipitated and ubiquitin was then immunoblotted; **d**, **e** c-Kit expression in Eca109 and KYSE-150 cells were analyzed after incubation with CHX. C-Kit was downregulated only when SOCS6 was overexpressed; **g**, **h** in Eca109 and KYSE-150 cells, c-Kit was immunoprecipitated and ubiquitin was then immunoblotted. The ubiquitylation level of c-Kit was decreased when SOCS6 was overexpressed; **f**, **i** SOCS6 and c-Kit were coimmunoprecipitated followed by WB. The data are presented as mean ± SD, *P < 0.05, **P < 0.01 and ***P < 0.001

The stem cell factor receptor c-Kit, an RTK, is deregulated in many cancers. Excessive c-Kit signaling primarily results in various cancers such as leukemia and tumors of the gastrointestinal tract and germ cells. Through UbiBrowser analysis and reviewing literature, we found that c-Kit can be ubiquitylated by SOCS6 and degraded through the ubiquitin proteasome system. Therefore, we speculated that degradation of c-Kit might explain the loss of ESCC cell stemness. Immunofluorescence signals analyzed by laser scanning confocal microscopy showed that SOCS6 and c-Kit were colocalized in cytoplasm (Fig. 6a). WB results showed that c-Kit expression decreased with SOCS6 overexpression (*P* < 0.001), indicating that the expression of c-Kit could be regulated by SOCS6 (Fig. 6b). HPV^+^ Eca109 cells were treated with MG132 (20 µg/mL) for 4 h, and then endogenous c-Kit was immunoprecipitated, and the level of ubiquitylation was monitored by immunoblotting. Overexpression of SOCS6 significantly promoted the ubiquitylation of c-Kit (Fig. 6c, *P* < 0.01). The mRNA level of c-Kit decreased slightly after SOCS6 overexpression (Additional file 3: Fig. S3, Eca109: *P* < 0.05, KYSE-150: *P* < 0.01). Cycloheximide (CHX) was used to inhibit new protein production before WB of c-Kit. Results showed that SOCS6 could accelerate c-Kit degradation (Fig. 6d, e). Furthermore, CHX chase assay was performed to examine the degradation rate of c-Kit with or without SOCS6. Results showed that SOCS6 could significantly accelerate the degradation of c-Kit in both Eca109 and KYSE-150 cells (Additional file 4: Fig. S4, *P *< 0.001). These results confirmed that c-Kit expression is regulated mostly at protein level, not mRNA level. To examine whether c-Kit could be ubiquitinated via SOCS6 in ESCC cells, c-Kit was immunoprecipitated, and the level of ubiquitylation was monitored by immunoblotting. Overexpression of SOCS6 in both Eca109 and KYSE-150 cells significantly increased the level of c-Kit ubiquitylation (Fig. 6 g, h, *P* < 0.001; *P* < 0.05). Moreover, IP of c-Kit pulled down endogenous SOCS6 protein from Eca109 cells and vice versa (Fig. 6f, i). Thus, SOCS6 targets c-Kit and promotes its ubiquitylation and degradation.

### SOCS6 inhibits tumor growth and sensitizes ESCC tumors to radiation in xenograft animal models

**Fig. 7:**
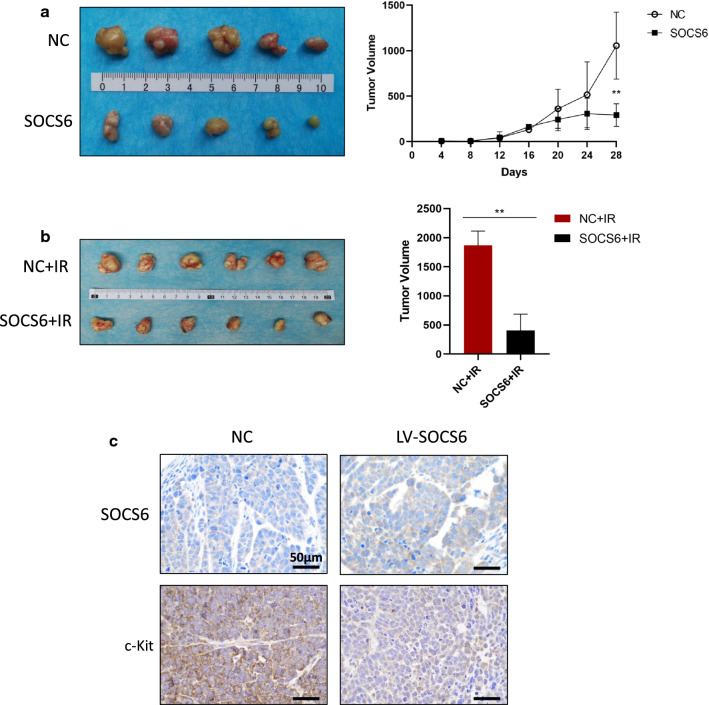
SOCS6 inhibits tumor growth and sensitizes ESCC tumors to radiation in xenograft animal models. **a** Eca109 cells expressing SOCS6 and control Eca109 cells were subcutaneously injected into nude mice (n = 5/group). The tumor volume was monitored every 4 days for 28 days. SOCS6 significantly inhibited tumor growth; **b** other mice were treated with radiation (2 Gy) when the tumor volume reached 100 mm^3^. The tumor size was measured 2 weeks later, and the tumor volume in the Eca109-SOCS6 group was decreased compared with that in the control group (n = 6/group). The statistical results are shown on the right. *IR* irradiation; **c** c-Kit was downregulated in tumors overexpressing SOCS6, as determined by IHC staining. The data are presented as mean ± SD, *P < 0.05, **P < 0.01 and ***P < 0.001

To investigate the in vivo effect of SOCS6 overexpression on tumor growth, we performed xenograft experiments in an animal model. Eca109 cells expressing SOCS6 and control Eca109 cells were subcutaneously injected into nude mice. The tumor volume was monitored every 4 days for 28 days. SOCS6 overexpression significantly slowed tumor growth in vivo (Fig. 7a, *P* < 0.01). Another group of mice were treated with radiation (2 Gy) when the tumor volume reached 100 mm^3^, and the tumor size was measured 2 weeks later. The volume of xenografts in the Eca109-SOCS6 group was lower than that in the control group (Fig. 7b, *P* < 0.01). Tumors were excised and subjected to IHC staining for SOCS6 and c-Kit proteins. C-Kit was downregulated in tumors overexpressing SOCS6 (Fig. 7c). These results demonstrate that overexpression of SOCS6 can inhibit ESCC tumor growth and sensitize ESCC cells to radiation.

## Discussion

Despite advances in radiation techniques and multimodal treatments, most ESCC patients develop acquired radioresistance, resulting in treatment failure. The radioresistance of ESCC urgently requires a solution. We previously reported that altered expression of SOCS6 by overexpression of PTENP1 reduced ESCC cell proliferation [37]. SOCS6 was first described to inhibit insulin receptor, which mediates cytokine-induced insulin resistance. The tumor suppression of SOCS6 was discovered in lung cancer, cervical cancer, gastric cancer and other types of cancers [16, 18, 19]. Here we observed that SOCS6 expression was decreased in ESCC tissue and higher expression of SOCS6 is related to more favorable prognosis. Therefore, we investigated the role of SOCS6 in ESCC malignancy. Survival analysis of 57 ESCC patients showed that SOCS6 is correlated with better prognosis. ESCC cell lines with overexpression or knock-down of SOCS6 were established, and HPV^+^ ESCC cells overexpressing SOCS6 were also constructed. Results showed that SOCS6 can sensitize HPV^+^ ESCC cells to radiation as well as in HPV negative ESCC cells. NHEJ and HR related proteins were decreased after overexpressing SOCS6. We found that the SOCS6 protein suppressed ESCC cell growth, migration and promoted cell sensitivity to cisplatin. These results were confirmed in vivo in nude mouse xenograft models. Since radioresistance correlates with CSCs, ESCC cell stemness was decreased by SOCS6 protein expression, as revealed by sphere formation and flow cytometric analyses. The loss of CSCs properties may explain the increased sensitivity to cisplatin and radiation. As a component of CRL5 E3 ubiquitin ligase complex, SOCS6 was found to interact with c-Kit and promote its ubiquitylation. These findings support the idea that SOCS6 is a promising therapeutic target to overcome radioresistance in ESCC patients.

The number of CSCs and their inherent radioresistance are important predictors of local control after radiotherapy [39]. CSC radioresistance is mediated by excessive activation of the two serine-threonine protein kinases in the DNA repair process, namely, ataxia-telangiectasia mutated (ATM) and ATM- and RAD3-Related (ATR) [40]. In addition to radiosensitivity, EMT and metastasis are closely linked to the CSC phenotype. Major transcription factors of the EMT signaling cascade, such as Snail, ZEB1, and Twist 1, have been proven to enhance cell stemness properties [41, 42]. In this work, flow cytometric analyses and sphere formation assays showed that SOCS6 protein expression sensitizes ESCC cells to radiation and cisplatin by reducing the population of CSCs.

Radiation is known to induce CSCs [30]. Cahu et al. suggested that CSCs appear after genotoxic stress due to recruitment of CSCs by chemokines secreted by senescent cells [43]. In breast cancer, radiation increased mammosphere formation and tumorigenicity by reprogramming differentiated breast cancer cell. Reprogramming resulted in the re-expression of several stem cell related genes, including Oct4, sex determining region Y-box 2, Nanog and Klf4 [44]. Zhang et al. reported the induction of dedifferentiation via releasing HMGB1 after X-ray irradiation [45]. Radiation also induces CSCs biomarker expression. CD44^+^ cell population increased after radiation in prostate cancer patients [46]. CD133 expression increased after 20 Gy of X-rays irradiation in pancreatic cancer [45]. However, the induction of CD271, CD24 and CD44 in ESCC after radiation has not been investigated. Our results show that radiation can significantly increase the population of CD271^+^ cell in Eca109 cells and that SOCS6 can counteract this induction partly.

The antitumor role of SOCS6 is reported to be associated mostly with its ability to downregulate JAK/STAT and PI3K/Akt signaling [47–49]. The ubiquitin-proteasome system is a major system that regulates protein turnover. One of the largest ubiquitin E3 ligase families is the Cullin-RING ubiquitin-protein ligase (CRL) family. SOCS6 has been reported to be a component of the CRL5 E3 ubiquitin ligase complex, which promotes Sin1 degradation and regulates cell survival and tumorigenesis [28]. Research has also shown that SOCS6 negatively regulates Flt3 signal transduction through direct interaction with phosphorylated tyrosines 591 and 919 in Flt3 and promotes Flt3 degradation [25]. C-Kit is an RTK associated with the stem cell niche. Gain-of-function mutations in c-Kit can promote tumor formation and progression in GIST, AML, mast cell leukemia and melanoma [47]. SOCS6 was predicted to target the juxtamembrane region of c-Kit, which affects MAPK activation [50]. The enhancing effect of c-Kit signaling on cell proliferation, combined with its link to stem cells, prompted us to explore methods to repress the expression of c-Kit or degrade it. This study showed that SOCS6 promotes the ubiquitylation and degradation of c-Kit, which may be the mechanism underlying the decrease in CSC properties in ESCC cells overexpressing SOCS6.

HPV infection is common in ESCC patients. Although the etiology has not been fully elucidated, HPV positivity is correlated with poor survival. The HPV E6 oncoprotein interferes with cell cycle regulation by binding and degrading the p53 tumor suppressor [51, 52]. In our previous study, we found that HPV^+^ ESCC cells are more resistant to radiation and we demonstrated a novel mechanism by which E6/E7 proteins enhance the stemness of ESCC cells [38]. In this study, we showed that SOCS6 can sensitize HPV^+^ ESCC cells to radiation and also analyzed SOCS6-induced changes in CSC properties in HPV^+^ ESCC cells. SOCS6 suppressed the induction of CSC properties by HPV infection, which is encouraging. Further mechanistic studies are ongoing.

However, the current study has certain limitations. How does SOCS6 overexpression caused the downregulation of HR and NHEJ signaling is still not clear. Interactions among HR, NHEJ and SOCS6 should be examined. The downstream signals of c-Kit which contributed to the promotion of radiosensitivity and suppression of CSCs population still need further investigation. Additionally, it is interesting that c-Kit expression is higher in HPV^+^ ESCC cell than HPV^−^ ESCC cells. These issues above are worthy of further investigations.

## Conclusions

In conclusion, our study identified a new mechanism underlying the radiosensitizing effect of the SOCS6 protein in ESCC. These findings have implications for clinical efforts to overcome radioresistance in ESCC patients.

## Supplementary Information

**Additional file 1: Figure S1.** SOCS6 expression is reduced in ESCC tissue. Differential expression of SOCS6 in ESCC tissues and normal esophageal tissues were analyzed in Oncomine database. (a) “Hu Esophagus” and (b) “Su Esophagus 2” datasets show that SOCS6 expression in ESCC tissue is lower than that in normal esophageal tissue. 0, normal esophageal tissue; 1, ESCC tissue.

**Additional file 2: Figure S2.** SOCS6 reverses the induction of stem cell properties by radiation. (a) Eca109 cells with or without 8 Gy of radiation were subjected to flow cytometry. CD271 expression was downregulated by SOCS6 after irradiation (IR); (b) the statistical results of flow cytometry are shown (n = 3). The data are presented as mean ± SD, *P* < 0.05 (*), *P* < 0.01 (**) and *P* < 0.001 (***).

**Additional file 3: Figure S3.** SOCS6 decreases *c-kit* expression. Total mRNA of Eca109 and KYSE-150 cells was extracted and RT-qPCR were performed to assess *c-kit* expression. The data are presented as mean ± SD, *P* < 0.05 (*), *P* < 0.01 (**).

**Additional file 4: Figure S4.** SOCS6 promoted the degradation of c-Kit. CHX were added to Eca109 (a) and KYSE-150 (b) cells to inhibit new protein production. Cells were collected and subjected to Western blotting at indicated time. The data are presented as mean ± SD, *P* < 0.001 (***).

## Data Availability

The datasets used and/or analysed during the current study are available from the corresponding author on reasonable request.

## Abbreviations

ESCC: Esophageal squamous cell carcinoma
CSCs: Cancer stem cells
CoIP: Coimmunoprecipitation
EAC: Esophageal adenocarcinoma
RT: Radiotherapy
OS: Overall survival
HPV: Human papillomavirus
HNSCC: Head and Neck Squamous Cell Carcinoma
SOCS: Suppressor of cytokine signaling
RTK: receptor tyrosine kinase
IRS4: Insulin receptor substrate 4
Flt3: FMS-like tyrosine kinase 3
UPS: Ubiquitin-proteasome system
AML: Acute myelogenous leukemia
GIST: Gastrointestinal stromal tumor
shRNA: Short hairpin RNAs
OD: Optical density
SF: Survival fraction
IP: Immunoprecipitation
IHC: Immunohistochemical
WB: Western blotting
NHEJ: Nonhomologous end-joining
HR: Homologous recombination
CHX: Cycloheximide
