# Supplementary figures and images for: SOCS6 promotes radiosensitivity and decreases cancer cell stemness in esophageal squamous cell carcinoma by regulating c-Kit ubiquitylation
Source: Cancer Cell Int. 2021 Mar 12;21:165. doi: 10.1186/s12935-021-01859-2 (PMC7953756; doi:10.1186/s12935-021-01859-2)

a

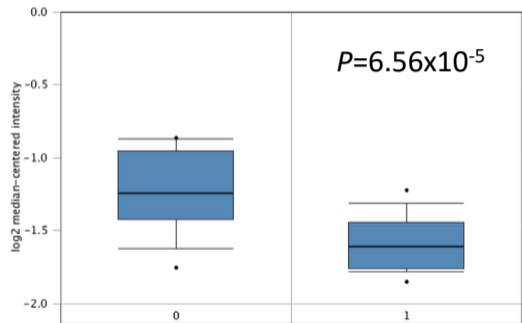

b

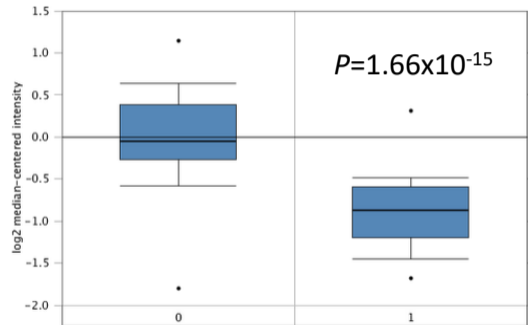

Supplement: Supplementary file 1 — Additional file 1: Figure S1. SOCS6 expression is reduced in ESCC tissue. Differential expression of SOCS6 in ESCC tissues and normal esophageal tissues were analyzed in Oncomine database. (a) “Hu Esophagus” and (b) “Su Esophagus 2” datasets show that SOCS6 expression in ESCC tissue is lower than that in normal esophageal tissue. 0, normal esophageal tissue; 1, ESCC tissue. [file 12935_2021_1859_MOESM1_ESM.pdf]

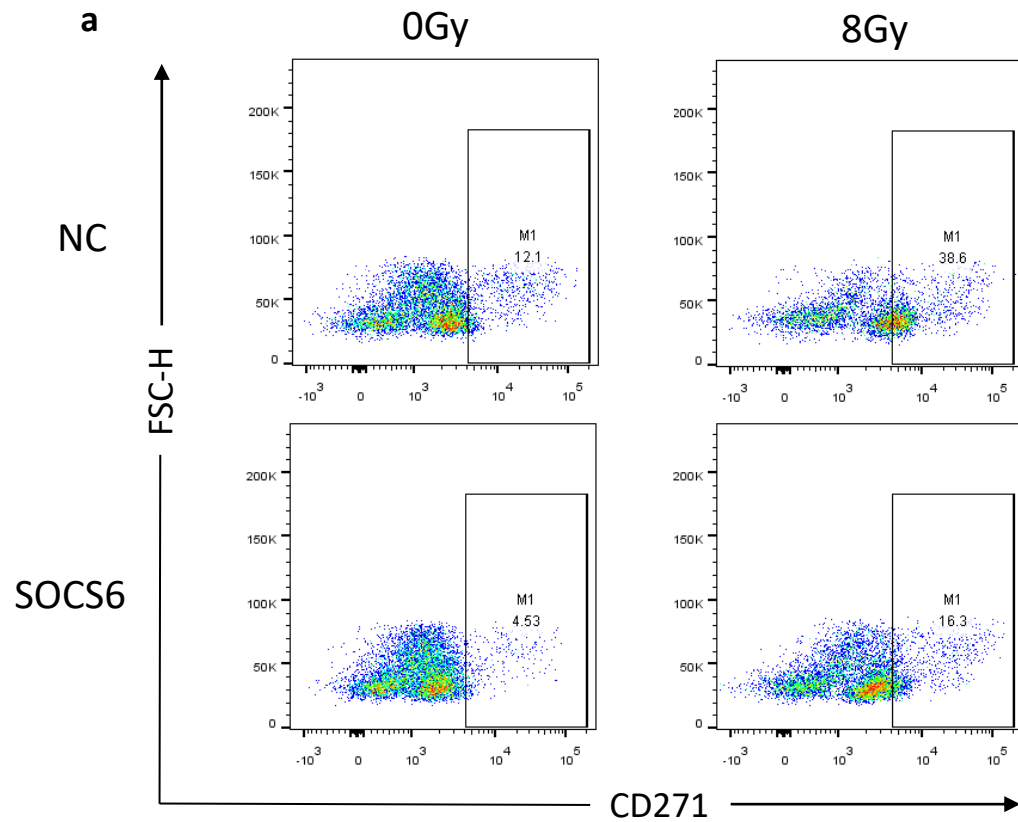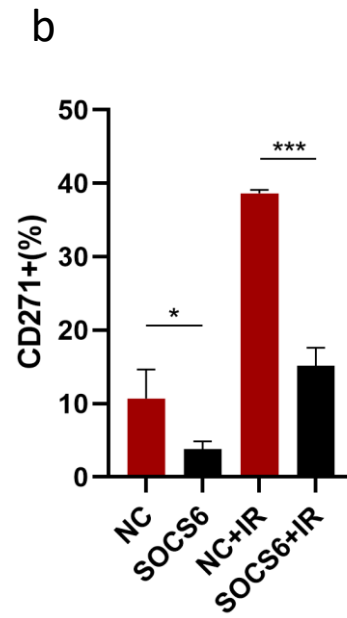

Supplement: Supplementary file 2 — Additional file 2: Figure S2. SOCS6 reverses the induction of stem cell properties by radiation. (a) Eca109 cells with or without 8 Gy of radiation were subjected to flow cytometry. CD271 expression was downregulated by SOCS6 after irradiation (IR); (b) the statistical results of flow cytometry are shown (n = 3). The data are presented as mean ± SD, P < 0.05 (*), P < 0.01 (**) and P < 0.001 (***). [file 12935_2021_1859_MOESM2_ESM.pdf]

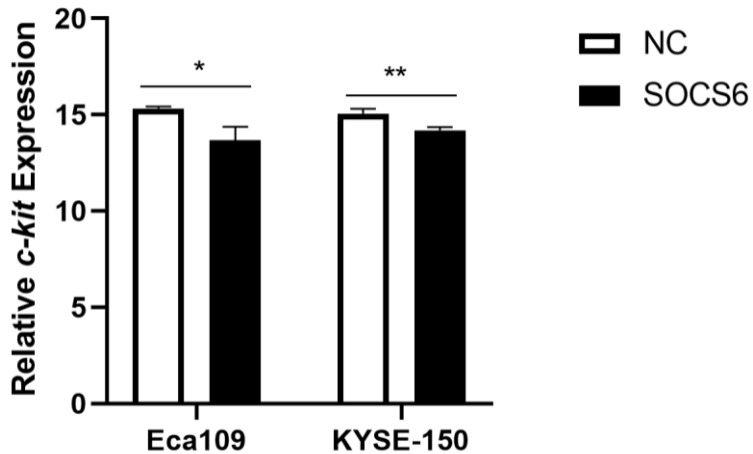

Supplement: Supplementary file 3 — Additional file 3: Figure S3. SOCS6 decreases c-kit expression. Total mRNA of Eca109 and KYSE-150 cells was extracted and RT-qPCR were performed to assess c-kit expression. The data are presented as mean ± SD, P < 0.05 (*), P < 0.01 (**). [file 12935_2021_1859_MOESM3_ESM.pdf]

**a**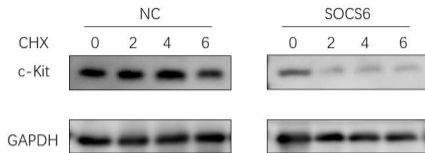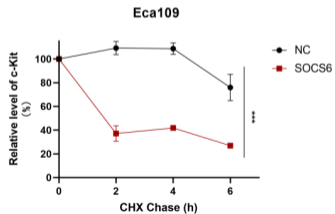**b**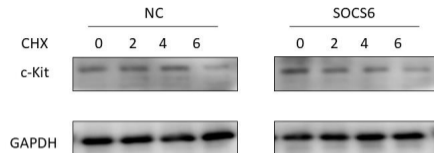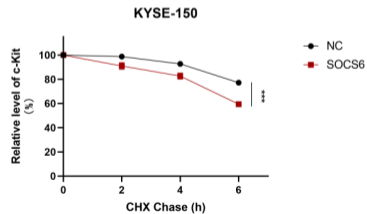

Supplement: Supplementary file 4 — Additional file 4: Figure S4. SOCS6 promoted the degradation of c-Kit. CHX were added to Eca109 (a) and KYSE-150 (b) cells to inhibit new protein production. Cells were collected and subjected to Western blotting at indicated time. The data are presented as mean ± SD, P < 0.001 (***). [file 12935_2021_1859_MOESM4_ESM.pdf]
